# Slowing gait during turning: how volition of modifying walking speed affects the gait pattern in healthy adults

**DOI:** 10.3389/fnhum.2024.1269772

**Published:** 2024-02-29

**Authors:** Julian Madrid, Leo Benning, Mischa Selig, Baptiste Ulrich, Brigitte M. Jolles, Julien Favre, David H. Benninger

**Affiliations:** ^1^Department of Clinical Neurosciences (DNC), Clinic of Neurology, Centre Hospitalier Universitaire Vaudois (CHUV), University of Lausanne (UNIL), Lausanne, Switzerland; ^2^University Emergency Center, Medical Center - University of Freiburg, Freiburg, Germany; ^3^Faculty of Medicine, University of Freiburg, Freiburg, Germany; ^4^Department of Orthopedics and Trauma Surgery, G.E.R.N. Research Center for Tissue Replacement, Regeneration and Neogenesis, Freiburg, Germany; ^5^Swiss BioMotion Lab, Department of Musculoskeletal Medicine (DAL), Centre Hospitalier Universitaire Vaudois (CHUV) and University of Lausanne (UNIL), Lausanne, Switzerland; ^6^Ecole Polytechnique Fédérale de Lausanne (EPFL), Institute of Microengineering, Lausanne, Switzerland

**Keywords:** gait, walking speed, volition, slowing, turning, cadence, variability, pace gait constant

## Abstract

**Background:**

Turning during walking and volitionally modulating walking speed introduces complexity to gait and has been minimally explored.

**Research question:**

How do the spatiotemporal parameters vary between young adults walking at a normal speed and a slower speed while making 90°, 180°, and 360° turns?

**Methods:**

In a laboratory setting, the spatiotemporal parameters of 10 young adults were documented as they made turns at 90°, 180°, and 360°. A generalized linear model was utilized to determine the effect of both walking speed and turning amplitude.

**Results:**

Young adults volitionally reducing their walking speed while turning at different turning amplitudes significantly decreased their cadence and spatial parameters while increasing their temporal parameters. In conditions of slower movement, the variability of certain spatial parameters decreased, while the variability of some temporal parameters increased.

**Significance:**

This research broadens the understanding of turning biomechanics in relation to volitionally reducing walking speed. Cadence might be a pace gait constant synchronizing the rhythmic integration of several inputs to coordinate an ordered gait pattern output. Volition might up-regulate or down-regulate this pace gait constant (i.e., cadence) which creates the feeling of modulating walking speed.

## Highlights

- Volitionally reducing walking speed decreases cadence for all turning amplitudes in young adults.
- Volitionally reducing walking speed while turning decreases spatial parameters in young adults.
- Volitionally reducing walking speed while turning increases temporal parameters in young adults.
- Volitionally reducing walking speed while turning decreases spatial parameter's variability and increases temporal parameter's variability in young adults.
- Cadence might be a pace gait constant which is volitionally controlled to regulate the walking speed.

## Introduction

Turning during walking represents a large amount of daily walking activity (Glaister et al., 2007). However, mostly straight-line walking has been investigated until now. Therefore, it is necessary to gain a more in-depth understanding of the gait pattern during turning in healthy subjects.

The turning amplitude is defined as the turning angle through which the subject changes its direction. Understanding the nuances of turning amplitudes can help in diagnosing and treating various health conditions. For instance, changes in turning amplitude can be indicative of aging, mobility issues, functional dependence in older adults, and neurological disorders like Parkinson's disease (Gill et al., 1995; Spildooren et al., 2010; Madrid and Benninger, 2021; Madrid et al., 2023). This knowledge can guide the development of personalized rehabilitation programs. In order to enable future studies on pathologic gait during turning, a solid understanding of turning in healthy subjects is needed.

Many studies investigated the biomechanical differences between self-selected natural walking speed and volitionally selected walking speed (fast, slow) during straight-line walking in healthy subjects (Kirtley et al., 1985; Riley et al., 2001; Grieve and Gear, 2007; Jordan et al., 2007; Roberts et al., 2017; Fukuchi et al., 2019; Winiarski et al., 2019), but solely few studies investigated this differences during turning (Akram et al., 2010; Fino and Lockhart, 2014; Forsell et al., 2017; Yamaguchi et al., 2017; Khobkhun et al., 2021). Furthermore, these studies focused mainly on the complex inter-segmental coordination of the head, trunk and lower limbs during turning at different speeds; an investigation of the spatiotemporal parameters during turning at different turning amplitudes and speeds is lacking. When investigating the effect of walking speed on gait, it is important to understand the relationship between walking speed, stride length and cadence: walking speed is modulated either by stride length and by cadence (Egerton et al., 2011). Although walking speed varies across age, turning amplitude, and a dual task, cadence seems to stay very stable (Smith et al., 2016; Madrid et al., 2023). Hence, in these conditions, walking speed may be modulated by stride length. Furthermore, walking speed varies when volitionally modulating it and in severe gait disorder such as progressive supranuclear palsy (PSP): in these two conditions –in contrast to the other ones– cadence is a major contributor to walking speed modulation (Winiarski et al., 2019; Ali et al., 2021).

There is a need to understand how healthy adults adapt their spatiotemporal gait pattern, particularly cadence and stride length to different turning conditions when volitionally modulating their walking speed. This interaction between volitionally slowing gait speed and turning at different amplitudes is the object of this study. In contrast to slow walking, fast walking may increase difficulty during turning. Hence, it would not be possible to differentiate the effect of volitionally modulating the walking speed and the effect of increased difficulty on spatiotemporal parameters. Therefore, we investigated the spatiotemporal parameters of young healthy adults volitionally reducing their gait speed at different turning amplitudes.

Furthermore, given that the variability of spatiotemporal parameters has been demonstrated to represent a good predictor of cognitive state and motor behavior (Lord et al., 2011), there is an interest in evaluating the effects of walking speed and turning amplitude on the variability of these parameters. Over the past decade, the variability of gait parameters has shown greater sensitivity in distinguishing healthy aging and pathological gait patterns than standard spatiotemporal parameters (Lord et al., 2011; Gaßner et al., 2020; Ma et al., 2020).

The objective of this study was to offer an exhaustive description and comparison of the spatiotemporal parameters, along with their variabilities, when young adults volitionally reduce their speed or maintain their self-selected normal speed while executing turns at 90°, 180°, and 360°. Specifically, this study examined the hypothesis that young adults, when voluntarily reducing their speed during 90°, 180°, and 360° turns compared to turns at their self-selected speed, modify their spatiotemporal gait patterns.

## Methods

### Participants

Participants for the study were recruited from a pool of healthy young adults aged between 20 and 30 years. Those with walking impairments or comorbidities that could affect gait, as well as those suffering from cardiac, respiratory, metabolic, neurological, or musculoskeletal pathologies, were not included in the study. Additionally, volunteers with obesity (BMI >30 kg/m^2^) were also excluded. The study group consisted of 10 young adults (five males, average age 23 ± 1 years, age range 21–24 years, BMI 21.3 ± 2.2 kg/m^2^). Given a sample size of 10 subjects and analyzing the step length of the external leg between normal and slow subjects, the study was sufficiently powered (>80%) to detect an effect size that was Cohen's *d* = 0.22 or larger. All participants provided written informed consent prior to their participation in the study, which received approval from the local ethics committee. This study is based on the same experiment performed to validate the algorithm detecting spatiotemporal parameters during turning (Ulrich et al., 2019). Furthermore, this study is a follow-up study built on the experimental procedure described in Madrid et al. (2023). We asked the healthy young participants of the previous study to turn at a self-selected reduced speed and compared it to the young participants turning at normal speed. Hence, instead of comparing the spatiotemporal parameters during turning at different amplitudes between older participants and young participants, we compared the spatiotemporal parameters during turning at different amplitudes between young participants, volitionally reducing their walking speed, and young participants walking at normal speed. The data of young participants walking at normal speed, which represented the control group, was used in both studies.

### Procedure

Participants were fitted with reflective markers on their feet and pelvis, as described by Ulrich et al. (2019). Markers were positioned bilaterally on the posterior side of the calcaneus and the second metatarsal heads. On the pelvis, markers were placed on both the anterior superior and posterior superior iliac spines. Following this, participants were instructed to carry out practice trials and the recorded turning trials, without footwear, in a laboratory outfitted with a motion capture system that recorded at a sampling rate of 120 Hz (Vicon, Oxford, UK).

**Figure 1:**
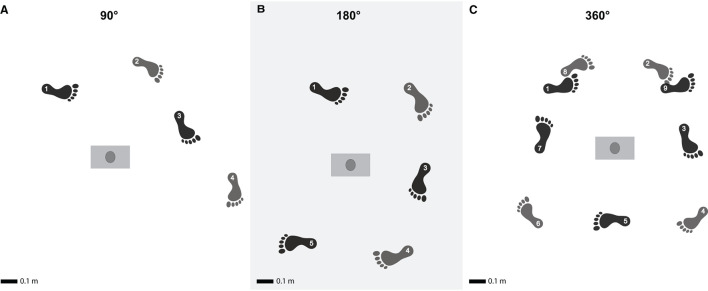
Turning at different turning amplitudes. Illustration of the experimental procedure representing the three turns during the experiment, with turns of 90° **(A)**, 180° **(B)** and 360° **(C)**. The colors indicate internal vs. external foot placement. The numbers indicate the order of the foot placements and start at turning initiation. Adapted from Madrid et al. (2023) with permission.

Participants were directed to walk in a straight line for 5 m, then make a 90°, 180°, or 360° turn around a 153 cm vertical rod, and subsequently continue walking straight for a minimum of 3 meters until they arrived at a predetermined point in the room, which was specific to the turn executed (Figure 1). There were no strict guidelines regarding the path the participant should take or whether the turn should be sharp or smooth. Therefore, we did not control for the turning radius, which could have influenced the turning strategy. For each turning amplitude, participants were requested to walk at their self-selected normal speed and at a speed slower than their self-selected normal speed. In contrast to slow walking, fast walking could potentially increase the difficulty of turning. Therefore, it would not have been possible to differentiate the effect of volitionally modulating the walking speed and the effect of increased difficulty on spatiotemporal parameters. For this reason, we did not test participants at speeds faster than their self-selected speed. The sequence of the turning amplitudes, walking speed conditions, and the direction of the turn (left or right) were randomized using a simple global randomization method. The direction of the turn was randomly chosen for each participant, who then performed all their trials maintaining the same turning direction. For each participant, nine trials were recorded for each walking speed condition and turning amplitude. Based on prior research, nine trials per condition seem to be a good tradeoff between sufficient data and unwanted effects such as fatigue (Madrid et al., 2023). The decision to include 10 participants per condition was informed by previous research on spatiotemporal parameters with similar descriptive and exploratory objectives (Bovonsunthonchai et al., 2014).

### Data processing

**Figure 2:**
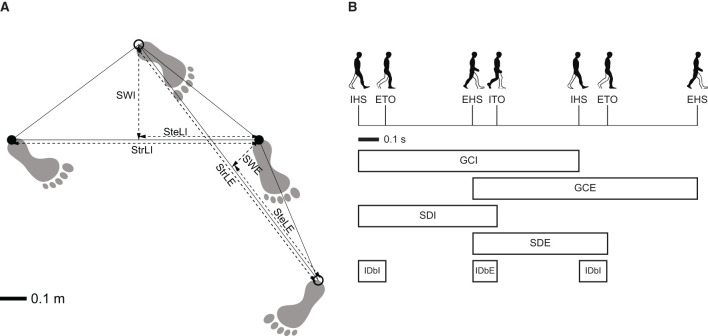
Definition of the spatiotemporal parameters. **(A)** Spatial gait parameters: stride length of the internal leg (StrLI); stride length of the external leg (StrLE); step length of the internal leg (SteLI); step length of the external leg (SteLE); stride width of the internal leg (SWI); stride width of the external leg (SWE). **(B)** Temporal gait parameters: internal heel-strike event (IHS); external toe-off event (ETO); external heel-strike event (EHS); internal toe-off event (ITO); gait cycle duration of internal leg (GCI); gait cycle duration of external leg (GCE); stance duration of internal leg (SDI); stance duration of external leg (SDE); initial double-support duration of internal leg (IDbI); initial double-support duration of external leg (IDbE). Adapted from Madrid et al. (2023) with permission.

Events of heel-strike and toe-off were identified using foot marker trajectories, adhering to the recommendations proposed by Ulrich et al. (2019). Specifically, these gait events were calculated using versions of the algorithm suggested by O'Connor et al. (2007) for heel-strike and Zeni et al. (2008) for toe-off, both of which have been validated for turning. The turning period was defined as the segment of the trials where the angular walking speed of the pelvis surpassed 30°/s (Mellone et al., 2016). Based on this definition, spatiotemporal parameters were analyzed from the last toe-off prior to the onset of the turning period to the first heel strike following the end of the turning period. The computed spatiotemporal parameters included: (1) for the entire turn: walking speed, total duration, number of steps, and cadence, (2) for the external and internal legs during each cycle: stride length, step width, step length, gait cycle duration, stance duration, stance/cycle ratio, and initial double-support duration (Figure 2) (Perry and Slac Thorofare, 1992; Huxham et al., 2006). The external leg was defined as the one exterior to the turning cycle, while the internal leg was defined to be interior to the turning cycle.

### Statistical analysis

Frequentist statistic methods, especially *p*-value-null-hypothesis-significance-testing (NHST), face a reproducibility crisis (Makowski et al., 2019; Keysers et al., 2020). The Bayesian framework solves many of these problems in an intuitive manner (Kruschke and Liddell, 2018). Notably, it enables the discrimination between the evidence of absence and the absence of evidence of an effect which is not possible in a frequentist framework (Keysers et al., 2020). We think this approach is particularly well suited to quantify knowledge and ignorance about clinical effects regarding the available data. Therefore, we decided to implement the Bayesian framework to perform inference (Dienes, 2014; Makowski et al., 2019; Keysers et al., 2020). Statistical analysis was performed using RStudio (v 1.2.1335, RStudio, Inc.).

For each participant, the measurements were aggregated across the nine trials of each turning amplitude. Subsequently, the mean and the within-subject standard deviation (SD), serving as an estimate of variability (Hausdorff et al., 2001; Galna et al., 2013), were computed for each aggregated dataset. This resulted in two data points (average value and variability) for each parameter, turning amplitude, and participant.

In order to ascertain whether walking speed and/or turning amplitude influenced the spatiotemporal parameters and their variability, statistical models were constructed separately for the mean and variability values of each parameter. These models incorporated a within-subject walking speed factor (normal walking speed or reduced walking speed) and a within-subject turning amplitude factor (90°, 180°, or 360°). This was achieved by performing 2 × 3 Bayesian repeated measures ANOVA and a Bayesian generalized linear model, with walking speed and turning amplitude as fixed effects and the subject's identification number as a random effect. Both walking speed and turning amplitude were treated as categorical variables.

We computed effect size estimates with 95% credible intervals and standardized effect sizes as Cohen's *d* (Cohen, 1988) or eta squared. In addition, we computed the Bayes factor (Dienes, 2014; Makowski et al., 2019), map-based *p*-values (Mills, 2018; Makowski et al., 2019) and the HDI+ ROPE method using the standardized effect size. The ROPE was calculated using a small effect size (0.2 standard deviations of all parameter data) as the region of practical equivalence (Kruschke, 2018). Hence, this study is not able to detect very small effect sizes. However we think they are not clinically significant (Cox, 2006).

We used residual plots and q-q plots to check the independence of observations, a non-explained trend in the residuals, linearity, normality, equality of variance and outliers. We investigated MCMC Convergence visually and using the Gelman diagnostic. We checked for Autocorrelation visually and used a minimum of 10,000 for the effective sample size of each parameter.

In the Bayesian framework, *p*-value corrections such as Bonferroni do not make much sense; therefore, we did not correct them (Berry and Hochberg, 1999; Gelman et al., 2012; Sjölander and Vansteelandt, 2019). Furthermore, even in a frequentist framework, *p*-value corrections are subject to debate (Rothman, 1990; Sjölander and Vansteelandt, 2019). We chose reasonably non-informative priors: we used a normal prior *N*(0;1.6) [it corresponds to a probability of 80% that the effect size lies between −2 and 2 (Gronau et al., 2020)]. We performed a Bayes factor robustness check visually to ensure that our prior had no significant influence on our posterior. To gain assurance about the accuracy of our complex model, we represented the actual data mean ± 95% CI statistics using bootstrapping and the model predictions graphically (Figures 2, 3 in Supplementary material 2, 4).

The significance level was set at a 95% credible Interval on the HDI ROPE of the standardized effect size for significance/equivalence testing.

## Results

For the sake of consistency, only statistically significant differences are presented in this section. In order to simplify the reading of the statistical results we used Cohen's *d* 95% confidence interval side which was nearest to zero. Therefore, the effects are always as high or higher when only one side of the confidence interval is given. When the effects are similar, the largest confidence interval is given on both sides. Therefore, the effects are always as small or smaller when the two sides of the confidence interval are given. A more standard presentation of the results with proper Cohen's *d* 95% CI is provided in Supplementary material 1, 3 and Tables 1, 2.

**Table 1:** Statistically significant ANOVA results (95% CI) are highlighted in green when there was an effect of velocity or turning amplitude, and in brown when there was a velocity × amplitude interaction.

**Parameter**	**YN mean (95% CI)**			**YS mean (95% CI)**			**Differences with respect to**	
	**90**°	**180**°	**360**°	**90**°	**180**°	**360**°	**Velocity**	**Turning amplitude**
Speed (m/s)	1.13 (1.10; 1.15)	0.99 (0.96; 1.01)	0.88 (0.86; 0.91)	0.88 (0.86; 0.91)	0.78 (0.75; 0.81)	0.72 (0.70; 0.75)	• 90°: YS < YN • 180°: YS < YN • 360°: YS < YN	• YN: 90°> 180°; 90°> 360°; 180°> 360° • YS: 90°> 180°; 90°> 360°; 180° > 360°
Turning duration (s)	1.46 (1.41; 1.51)	2.22 (2.16; 2.27)	3.58 (3.49; 3.65)	1.53 (1.47; 1.58)	2.49 (2.43; 2.56)	4.22 (4.08; 4.34)	• 90°: YS ≈ YN • 180°: YS ⇔ YN • 360°: YS > YN	• YN: 90° < 180°; 90° < 360°; 180° < 360° • YS: 90° < 180°; 90° < 360°; 180° < 360°
Number of steps (–)	3.75 (3.66; 3.86)	5.11 (5.00; 5.21)	7.71 (7.54; 7.86)	3.40 (3.29; 3.50)	4.84 (4.71; 4.94)	7.57 (7.41; 7.72)	• 90°: YS ⇔ YN • 180°: YS ⇔ YN • 360°: YS ≈ YN	• YN: 90° < 180°; 90° < 360°; 180° < 360° • YS: 90° < 180°; 90° < 360°; 180° < 360°
Cadence (steps/min)	113 (111; 114)	111 (109; 112)	112 (111; 113)	93 (91; 96)	92 (90; 95)	94 (92; 96)	• 90°: YS < YN • 180°: YS < YN • 360°: YS < YN	• YN: 90°≈ 180°; 90°≈ 360°; 180°≈ 360° • YS: 90° ⇔ 180°; 90°≈ 360°; 180°≈ 360°
Stride length: internal leg (m)	1.063 (1.037; 1.089)	0.927 (0.900; 0.954)	0.835 (0.818; 0.854)	0.954 (0.923; 0.984)	0.810 (0.786; 0.834)	0.746 (0.730; 0.759)	• 90°: YS < YN • 180°: YS < YN • 360°: YS < YN	• YN: 90°> 180°; 90°> 360°; 180°> 360° • YS: 90°> 180°; 90°> 360°; 180° ⇔ 360°
Stride length: external leg (m)	1.249 (1.233; 1.268)	1.119 (1.099; 1.142)	1.069 (1.055; 1.085)	1.121 (1.101; 1.143)	1.039 (1.020; 1.058)	1.000 (0.988; 1.011)	• 90°: YS < YN • 180°: YS < YN • 360°: YS < YN	• YN: 90°> 180°; 90°> 360°; 180°> 360° • YS: 90°> 180°; 90°> 360°; 180° ⇔ 360°
Step length: internal leg (m)	0.534 (0.517; 0.551)	0.454 (0.438; 0.473)	0.403 (0.392; 0.416)	0.484 (0.464; 0.506)	0.397 (0.381; 0.412)	0.359 (0.349; 0.369)	• 90°: YS < YN • 180°: YS < YN • 360°: YS < YN	• YN: 90°> 180°; 90°> 360°; 180°> 360° • YS: 90°> 180°; 90°> 360°; 180°> 360°
Step length: external leg (m)	0.642 (0.632; 0.652)	0.575 (0.561; 0.588)	0.550 (0.541; 0.561)	0.574 (0.560; 0.588)	0.530 (0.519; 0.543)	0.515 (0.508; 0.523)	• 90°: YS < YN • 180°: YS < YN • 360°: YS < YN	• YN: 90°> 180°; 90°> 360°; 180° ⇔ 360° • YS: 90° ⇔ 180°; 90°> 360°; 180° ⇔ 360°
Stride width: internal leg (m)	0.361 (0.346; 0.377)	0.357 (0.344; 0.370)	0.376 (0.369; 0.383)	0.360 (0.344; 0.376)	0.357 (0.349; 0.367)	0.367 (0.360; 0.373)	• 90°: YS ⇔ YN • 180°: YS ⇔ YN • 360°: YS ⇔ YN	• YN: 90° ⇔ 180°; 90° ⇔ 360°; 180° ⇔ 360° • YS: 90° ⇔ 180°; 90° ⇔ 360°; 180° ⇔ 360°
Stride width: external leg (m)	−0.0719 (−0.0873; −0.0555)	−0.136 (−0.153; −0.120)	−0.159 (−0.169; −0.149)	−0.0913 (−0.1134; −0.0685)	−0.116 (−0.132; −0.101)	−0.126 (−0.136; −0.118)	• 90°: YS ⇔ YN • 180°: YS ⇔ YN • 360°: YS ⇔ YN	• YN: 90° < 180°; 90° < 360°; 180° ⇔ 360° • YS: 90° ⇔ 180°; 90° ⇔ 360°; 180° ⇔ 360°
Gait cycle duration: internal leg (s)	1.04 (1.03; 1.06)	1.07 (1.06; 1.08)	1.06 (1.05; 1.07)	1.25 (1.22; 1.29)	1.30 (1.27; 1.32)	1.28 (1.26; 1.29)	• 90°: YS > YN • 180°: YS > YN • 360°: YS > YN	• YN: 90° ⇔ 180°; 90°≈ 360°; 180°≈ 360° • YS: 90° ⇔ 180°; 90°≈ 360°; 180° ⇔ 360°
Gait cycle duration: external leg (s)	1.07 (1.05; 1.08)	1.08 (1.07; 1.09)	1.07 (1.06; 1.07)	1.27 (1.23; 1.30)	1.29 (1.26; 1.32)	1.29 (1.27; 1.30)	• 90°: YS > YN • 180°: YS > YN • 360°: YS > YN	• YN: 90° ⇔ 180°; 90°≈ 360°; 180°≈ 360° • YS: 90° ⇔ 180°; 90° ⇔ 360°; 180° ⇔ 360°
Stance duration: internal leg (s)	0.655 (0.648; 0.663)	0.673 (0.665; 0.681)	0.668 (0.661; 0.674)	0.809 (0.793; 0.825)	0.833 (0.819; 0.847)	0.820 (0.809; 0.830)	• 90°: YS > YN • 180°: YS > YN • 360°: YS > YN	• YN: 90° ⇔ 180°; 90° ⇔ 360°; 180°≈ 360° • YS: 90° ⇔ 180°; 90°≈ 360°; 180° ⇔ 360°
Stance duration: external leg (s)	0.642 (0.635; 0.649)	0.653 (0.646; 0.660)	0.637 (0.632; 0.642)	0.784 (0.767; 0.801)	0.797 (0.782; 0.814)	0.788 (0.778; 0.797)	• 90°: YS > YN • 180°: YS > YN • 360°: YS > YN	• YN: 90°≈ 180°; 90°≈ 360°; 180° ⇔ 360° • YS: 90° ⇔ 180°; 90°≈ 360°; 180° ⇔ 360°
Initial double-support duration: internal leg (s)	0.119 (0.115; 0.123)	0.131 (0.127; 0.135)	0.127 (0.124; 0.129)	0.166 (0.159; 0.173)	0.178 (0.171; 0.184)	0.175 (0.171; 0.180)	• 90°: YS > YN • 180°: YS > YN • 360°: YS > YN	• YN: 90° ⇔ 180°; 90° ⇔ 360°; 180°≈ 360° • YS: 90° ⇔ 180°; 90° ⇔ 360°; 180°≈ 360°
Initial double- support duration: external leg (s)	0.108 (0.105; 0.111)	0.114 (0.111; 0.117)	0.112 (0.110; 0.114)	0.153 (0.147; 0.159)	0.161 (0.156; 0.165)	0.160 (0.156; 0.164)	• 90°: YS > YN • 180°: YS > YN • 360°: YS > YN	• YN: 90° ⇔ 180°; 90°≈ 360°; 180°≈ 360° • YS: 90° ⇔ 180°; 90° ⇔ 360°; 180°≈ 360°
Stance/cycle ratio : internal leg (%)	60.31 (59.99; 60.64)	61.79 (61.47; 62.09)	62.48 (62.25; 62.70)	62.47 (62.06; 62.82)	63.91 (63.61; 64.21)	64.39 (64.15; 65.64)	• 90°: YS > YN • 180°: YS > YN • 360°: YS > YN	• YN: 90° < 180°; 90° < 360°; 180° < 360° • YS: 90° < 180°; 90° < 360°; 180° < 360°
Stance/cycle ratio: external leg (%)	59.31 (58.83; 59.82)	59.54 (59.19; 59.91)	59.36 (59.12; 59.62)	60.74 (60.11; 61.34)	61.05 (60.59; 61.51)	61.33 (61.05; 61.62)	• 90°: YS > YN • 180°: YS > YN • 360°: YS > YN	• Both: 90°≈ 180°; 90° ⇔ 360°; 180° ⇔ 360° • Both: 90° ⇔ 180°; 90° ⇔ 360°; 180° ⇔ 360°

The absence of an effect is highlighted in blue and undecided results are highlighted in yellow.

YN, young adults with normal speed; YS, young adults with reduced speed; <, significantly smaller; >, significantly greater; ≈, equivalent (i.e. absence of an effect); ⇔, undecided; 90°, quarter-turn; 180°, half-turn; 360°, full-turn.

**Table 2:** Statistically significant ANOVA results (95% CI) are highlighted in green when there was an effect of velocity or turning amplitude, and in brown when there was a velocity × amplitude interaction.

**Parameter SD**	**YN mean (95% CI)**			**YS mean (95% CI)**			**Differences with respect to**	
	**90**°	**180**°	**360**°	**90**°	**180**°	**360**°	**Velocity**	**Turning amplitude**
SD speed (m/s)	0.0443 (0.0349; 0.0545)	0.042 (0.034; 0.052)	0.034 (0.029; 0.039)	0.0406 (0.033; 0.049)	0.035 (0.029; 0.042)	0.031 (0.025; 0.037)	• 90°: YS ⇔ YN • 180°: YS ⇔ YN • 360°: YS ⇔ YN	• YN: 90° ⇔ 180°; 90° ⇔ 360°; 180° ⇔ 360° • YS: 90° ⇔ 180°; 90° ⇔ 360°; 180° ⇔ 360°
SD turning duration (s)	0.206 (0.130; 0.284)	0.262 (0.207; 0.309)	0.278 (0.222; 0.368)	0.289 (0.248; 0.336)	0.292 (0.242; 0.346)	0.328 (0.250; 0.403)	• 90°: YS ⇔ YN • 180°: YS ⇔ YN • 360°: YS ⇔ YN	• YN: 90° ⇔ 180°; 90° ⇔ 360°; 180° ⇔ 360° • YS: 90° ⇔ 180°; 90° ⇔ 360°; 180° ⇔ 360°
SD number of steps (–)	0.367 (0.205; 0.528)	0.483 (0.363; 0.597)	0.471 (0.386; 0.594)	0.467 (0.392; 0.558)	0.458 (0.385; 0.544)	0.446 (0.332; 0.538)	• 90°: YS ⇔ YN • 180°: YS ⇔ YN • 360°: YS ⇔ YN	• YN: 90° ⇔ 180°; 90° ⇔ 360°; 180° ⇔ 360° • YS: 90° ⇔ 180°; 90° ⇔ 360°; 180° ⇔ 360°
SD cadence (steps/min)	2.88 (2.44; 3.37)	2.69 (2.27; 3.12)	3.16 (2.56; 3.66)	4.14 (3.13; 5.27)	3.108 (2.85; 3.55)	2.54 (1.94; 3.28)	• 90°: YS > YN • 180°: YS ⇔ YN • 360°: YS ⇔ YN	• YN: 90° ⇔ 180°; 90° ⇔ 360°; 180° ⇔ 360° • YS: 90° ⇔ 180°; 90° < 360°; 180° ⇔ 360°
SD stride length: internal leg (m)	0.0676 (0.0531; 0.0832)	0.165 (0.150; 0.177)	0.130 (0.115; 0.147)	0.0865 (0.0603; 0.1183)	0.120 (0.099; 0.142)	0.0985 (0.0847; 0.1173)	• 90°: YS ⇔ YN • 180°: YS < YN • 360°: YS ⇔ YN	• YN: 90° < 180°; 90° < 360°; 180° ⇔ 360° • YS: 90° ⇔ 180°; 90° ⇔ 360°; 180° ⇔ 360°
SD stride length: external leg (m)	0.0552 (0.0434; 0.0693)	0.102 (0.084; 0.122)	0.0994 (0.082; 0.117)	0.0481 (0.0396; 0.0572)	0.0794 (0.0664; 0.1092)	0.0778 (0.0663; 0.0916)	• 90°: YS ⇔ YN • 180°: YS ⇔ YN • 360°: YS ⇔ YN	• YN: 90° < 180°; 90° < 360°; 180° ⇔ 360° • YS: 90° < 180°; 90° < 360°; 180° ⇔ 360°
SD step length: internal leg (m)	0.048 (0.031; 0.071)	0.091 (0.078; 0.106)	0.084 (0.071; 0.097)	0.051 (0.038; 0.065)	0.080 (0.070; 0.092)	0.068 (0.059; 0.077)	• 90°: YS ⇔ YN • 180°: YS ⇔ YN • 360°: YS ⇔ YN	• YN: 90° < 180°; 90° < 360°; 180° ⇔ 360° • YS: 90° < 180°; 90° ⇔ 360°; 180° ⇔ 360°
SD step length: external leg (m)	0.037 (0.024; 0.050)	0.061 (0.049; 0.073)	0.058 (0.047; 0.070)	0.032 (0.026; 0.038)	0.047 (0.039; 0.055)	0.045 (0.039; 0.052)	• 90°: YS ⇔ YN • 180°: YS ⇔ YN • 360°: YS ⇔ YN	• YN: 90° < 180°; 90° < 360°; 180° ⇔ 360° • YS: 90° ⇔ 180°; 90° ⇔ 360°; 180° ⇔ 360°
SD stride width: internal leg (m)	0.058 (0.042; 0.075)	0.073 (0.061; 0.084)	0.047 (0.037; 0.057)	0.052 (0.042; 0.066)	0.049 (0.039; 0.059)	0.042 (0.035; 0.049)	• 90°: YS ⇔ YN • 180°: YS < YN • 360°: YS ⇔ YN	• YN: 90° ⇔ 180°; 90° ⇔ 360°; 180°> 360° • YS: 90° ⇔ 180°; 90° ⇔ 360°; 180° ⇔ 360°
SD stride width: external leg (m)	0.068 (0.056; 0.083)	0.085 (0.065; 0.10)	0.070 (0.057; 0.083)	0.071 (0.051; 0.094)	0.068 (0.043; 0.094)	0.059 (0.049; 0.070)	• 90°: YS ⇔ YN • 180°: YS ⇔ YN • 360°: YS ⇔ YN	• YN: 90° ⇔ 180°; 90° ⇔ 360°; 180° ⇔ 360° • YS: 90° ⇔ 180°; 90° ⇔ 360°; 180° ⇔ 360°
SD gait cycle duration: internal leg (s)	0.036 (0.031; 0.041)	0.052 (0.045; 0.057	0.054 (0.046; 0.061)	0.055 (0.042; 0.072)	0.067 (0.057; 0.078)	0.052 (0.041; 0.064)	• 90°: YS > YN • 180°: YS ⇔ YN • 360°: YS ⇔ YN	• YN: 90° < 180°; 90° < 360°; 180° ⇔ 360° • YS: 90° ⇔ 180°; 90° ⇔ 360°; 180° ⇔ 360°
SD gait cycle duration: external leg (s)	0.028 (0.022; 0.034)	0.036 (0.029; 0.043)	0.040 (0.034; 0.046)	0.059 (0.040; 0.079)	0.053 (0.043; 0.063)	0.044 (0.034; 0.057)	• 90°: YS > YN • 180°: YS ⇔ YN • 360°: YS ⇔ YN	• YN: 90° ⇔ 180°; 90° ⇔ 360°; 180° ⇔ 360° • YS: 90° ⇔ 180°; 90° ⇔ 360°; 180° ⇔ 360°
SD stance duration: internal leg (s)	0.033 (0.027; 0.039)	0.039 (0.034; 0.045)	0.040 (0.036; 0.044)	0.047 (0.035; 0.061)	0.052 (0.043; 0.061)	0.047 (0.039; 0.055)	• 90°: YS > YN • 180°: YS > YN • 360°: YS ⇔ YN	• YN: 90° ⇔ 180°; 90° ⇔ 360°; 180° ⇔ 360° • YS: 90° ⇔ 180°; 90° ⇔ 360°; 180° ⇔ 360°
SD stance duration: external leg (s)	0.024 (0.019; 0.029)	0.030 (0.025; 0.036)	0.031 (0.028; 0.034)	0.038 (0.028; 0.050)	0.040 (0.032; 0.048)	0.036 (0.029; 0.043)	• 90°: YS > YN • 180°: YS ⇔ YN • 360°: YS ⇔ YN	• YN: 90° ⇔ 180°; 90° ⇔ 360°; 180° ⇔ 360° • YS: 90° ⇔ 180°; 90° ⇔ 360°; 180° ⇔ 360°
SD Initial double- support duration: internal leg (s)	0.012 (0.009; 0.013)	0.016 (0.013; 0.020)	0.017 (0.016; 0.019)	0.020 (0.014; 0.027)	0.024 (0.019; 0.030)	0.022 (0.019; 0.026)	• 90°: YS > YN • 180°: YS > YN • 360°: YS ⇔ YN	• YN: 90° ⇔ 180°; 90° ⇔ 360°; 180° ⇔ 360° • YS: 90° ⇔ 180°; 90° ⇔ 360°; 180° ⇔ 360°
SD Initial double-support duration: external leg (s)	0.013 (0.011; 0.015)	0.015 (0.012; 0.017)	0.016 (0.014; 0.019)	0.016 (0.013; 0.020)	0.016 (0.013; 0.020)	0.019 (0.016; 0.021)	• 90°: YS ⇔ YN • 180°: YS ⇔ YN • 360°: YS ⇔ YN	• YN: 90° ⇔ 180°; 90° ⇔ 360°; 180° ⇔ 360° • YS: 90° ⇔ 180°; 90° ⇔ 360°; 180° ⇔ 360°
SD stance/cycle ratio: internal leg (%)	1.13 (0.92; 1.41)	1.44 (1.16; 1.72)	1.54 (1.31; 1.78)	1.15 (0.82; 1.60)	1.40 (1.27; 1.54)	1.42 (1.21; 1.63)	• 90°: YS ⇔ YN • 180°: YS ⇔ YN • 360°: YS ⇔ YN	• YN: 90° ⇔ 180°; 90° ⇔ 360°; 180° ⇔ 360° • YS: 90° ⇔ 180°; 90° ⇔ 360°; 180° ⇔ 360°
SD stance/cycle ratio: external leg (%)	1.41 (0.93; 1.92)	1.54 (1.27; 1.81)	1.47 (1.30; 1.72)	1.59 (1.11; 2.07)	1.55 (1.15; 2.02)	1.35 (1.14; 1.64)	• 90°: YS ⇔ YN • 180°: YS ⇔ YN • 360°: YS ⇔ YN	• YN: 90° ⇔ 180°; 90° ⇔ 360°; 180° ⇔ 360° • YS: 90° ⇔ 180°; 90° ⇔ 360°; 180° ⇔ 360°

The absence of an effect is highlighted in blue and undecided results are highlighted in yellow.

YN, young adults with normal speed; YS, young adults with reduced speed; <, significantly smaller; >, significantly greater; ≈, equivalent (i.e., absence of an effect); ⇔, undecided; 90°, quarter-turn; 180°, half-turn; 360°, full-turn.

### Spatiotemporal parameters

Participants walked significantly slower when they were asked to decrease their walking speed (Cohen's *d* < −2.05). Furthermore, in both speed conditions, young adults decreased their speed with larger turning amplitudes (Cohen's *d* < −0.5). Turning duration between conditions were similar at 90° (Cohen's *d* −0.29, 0.50) and differed at 360° (Cohen's *d* 1.46, 2.31). The total number of steps was similar at 360° (Cohen's *d* −0.64, 0.15). Cadence was significantly decreased in the slow-speed condition compared to the normal condition for all turning amplitudes (Cohen's *d* < −2.42). At normal speed, cadence was similar between all turning amplitudes (Cohen's *d* −0.33, 0.51) while at slower speed, cadence was similar between all turning amplitudes (Cohen's *d* −0.39, 0.31) except between 90° and 180°.

When participants were asked to decrease their speed, stride length and step length of the internal and external leg were significantly shorter (Cohen's *d* < -0.33). At normal speed and for both legs, there was a decrease in stride length with higher amplitude between 90° vs. 180° and 180° vs. 360° (Cohen's *d* < -0.33), whereas for the slower condition and for both legs stride length decreased with higher amplitude at 180° and 360° compared to 90° (Cohen's *d* < -0.32). The step length of the internal leg decreased with higher amplitude between 90° vs. 180° and 180° vs. 360° for the slower and normal conditions (Cohen's *d* < -0.25). At normal speed, the step length of the external leg decreased for the 180° and 360° turns compared to the 90° (Cohen's *d* < -0.69). At a slower speed, the step length of the external leg was decreased only in 360° amplitude compared to the 90° amplitude (Cohen's *d* < -0.54). For the normal condition, the stride width of the external leg decreased with higher amplitude between 90° vs. 180° and 90° vs. 360° (Cohen's *d* < -0.53).

Gait cycle duration, stance duration, initial double support and stance/cycle ratio of the internal and external legs of all turning amplitudes were increased at a slower speed compared to normal speed (Cohen's *d* >0.43).

The gait cycle durations of the internal leg were similar for 90° vs. 360° and 180° vs. 360° at normal speed (Cohen's *d* −0.41, 0.52) and for 90° vs. 360° at slower speed (Cohen's *d* −0.24, 0.51). The gait cycle durations of the external leg were similar for 90° vs. 360° and 180° vs. 360° at normal speed (Cohen's *d* −0.48, 0.30). The stance durations of the internal leg were similar for 180° vs. 360° at normal speed (Cohen's *d* −0.24, 0.20) and for 90° vs. 360° at lower speed (Cohen's *d* −0.09, 0.43). The stance durations of the external leg were similar for 90° vs. 180° and 90° vs. 360° at normal speed (Cohen's *d* −0.35, 0.45), and for 90° vs. 360° at slower speed (Cohen's *d* −0.32, 0.20). The initial double support durations for the internal leg were similar at 180° vs. 360° at both normal and slower speeds, respectively (Cohen's *d* −0.38, 0.20). The double support of the external leg was similar for 90° vs. 360° and 180° vs. 360° at normal speed (Cohen's *d* −0.28, 0.37), and for 180° vs. 360° at slower speed (Cohen's *d* −0.28, 0.10). The stance/cycle ratio of the internal leg increased significantly at larger turning amplitudes in both speed conditions (Cohen's *d* >0.18). The stance/cycle ratios of the external leg were similar at 90° vs. 180° at normal speed (Cohen's *d* −0.31, 0.39).

These results are summarized in Table 1 and Supplementary material 1, 2.

### Spatiotemporal parameters variability

Cadence's variability was significantly greater in the slower condition at 90° compared to the normal condition at 90° (Cohen's *d* >0.36) and significantly decreased between 90° and 360° for the slower condition (Cohen's *d* < −0.35).

The stride length and step width of the internal leg's variabilities were greater for the normal condition at 180° (Cohen's *d* < −0.37) compared to the slower condition. The stride length of the internal leg's variability increased for the normal condition between 90° vs. 180° and 90° vs. 360° (Cohen's *d* >1.04). The stride length of the external leg's variability increased for both conditions between 90° vs. 180° and 90° vs. 360° (Cohen's *d* >0.47). The step length of the internal leg's variability increased for the normal condition between 90° vs. 180° and 90° vs. 360° and for the slower condition between 90° vs. 180° (Cohen's *d* >0.42). The step length of the external leg's variability increased for the normal condition between 90° vs. 180° and 90° vs. 360° (Cohen's *d* >0.47). The step width of the internal leg's variability decreased for the normal condition between 180° and 360° (Cohen's *d* < −0.53).

The gait cycle duration of the internal leg and external leg variability was increased for the slower condition at 90° (Cohen's *d* >0.46) compared to the normal condition. The gait cycle duration of the internal leg's variability increased between 90° vs. 180° and 90° vs. 360° for the normal condition (Cohen's *d* >0.27). The stance duration of the internal leg's variability was significantly increased for the slower condition compared to the normal condition at 90° and 180° (Cohen's *d* >0.32) and the stance duration of the external leg's variability was significantly increased for the slower condition compared to the normal condition at 90° (Cohen's *d* >0.58). The initial double support duration of the internal leg's variability was significantly increased at 90° and 180° when participants were asked to decrease their speed (Cohen's *d* >0.31).

These results are summarized in Table 2 and Supplementary material 3, 4.

## Discussion

The spatiotemporal parameters during turning maneuvers exhibited statistical differences when young adults volitionally reduced their walking speed. When participants were asked to decrease their speed, they decreased their cadence, needed more time to turn at high turning amplitudes, and the stride and step lengths of the internal and external legs were decreased. Additionally, certain temporal parameters of the internal and external legs increased. However, the turning duration and the number of steps were similar in both conditions for 90° and 360° turns respectively. These findings broaden our comprehension of how gait differences relate to turning biomechanics when the walking speed is intentionally adjusted. They corroborate the findings of previous studies that examined a limited set of parameters, particularly in relation to cadence, stride length and gait cycle duration in adults decreasing their walking speed while straight line walking (Pietraszewski et al., 2012; Winiarski et al., 2019).

This study serves as a follow-up to address questions arising from the experiment conducted by Madrid et al. (2023). In their study, despite a significant reduction in velocity and spatial parameters among older healthy participants relative to their younger counterparts during turning, cadence remained similar across various aging conditions and turning amplitudes. We posited that cadence acts as the central integrative element between volitionally modulating walking speed and the output of complex gait patterns during turns. If this hypothesis holds, then young participants who voluntarily reduce their walking speed while turning should exhibit a decrease in cadence when compared to turning at a self-selected speed. This study confirmed that, indeed, cadence significantly decreased in young adults who volitionally reduced their walking speed under all turning conditions. The walking speed reduction in elderly adults is due to a reduction in spatial parameters, whereas the cadence stays constant (Madrid et al., 2023). As shown in this study, in young adults slowing their gait, the walking speed reduction is mostly due to a reduction in cadence. Furthermore, this relationship has also been shown for straight line walking (Egerton et al., 2011). Cadence might be a pace gait constant synchronizing the rhythmic integration of several inputs to coordinate an ordered gait pattern output. Volition might up-regulate or down-regulate this pace gait constant (i.e., cadence), which creates the feeling of modulating walking speed. The importance of cadence as a gait constant synchronizing the complex rhythmic integration of several inputs to coordinate an ordered gait pattern output might have important implications for diseases where this integration is impaired. Freezing of gait is believed to represent such a condition where the ordered gait pattern output is impaired (Gao et al., 2020); interestingly, volitionally focusing on cues that enhance the rhythmicity of gait has been reported to reduce freezing of gait occurrence (such as a rhythmic auditive cue or a repetitive visual cue on the floor) (Rutz and Benninger, 2020). Cadence might represent the essential link between the integration of such beneficial inputs and an ordered output. Furthermore, it would be interesting to test new physiotherapeutic strategies focusing on volitionally controlling cadence in order to improve gait in such pathologies. Further research on the relationship between cadence and gait pathologies, such as freezing of gait, is needed.

The brain seems to use oscillations to code and integrate information (Schyns et al., 2011). When these oscillations are perturbed, pathologies emerge called oscillopathies (Madrid and Benninger, 2021). Especially Parkinson's disease and freezing of gait are believed to show altered rhythmic oscillations. The rhythmic integration of input reflected in cadence might suffer from these altered oscillations. Further research is needed.

On average, both the adults in the slow walking speed and normal walking speed conditions used a crossover strategy (seen in the negative stride width of the external leg). A crossover strategy consists of an individual stepping across their internal leg (Bhatt et al., 2013). Furthermore, in both walking speed conditions, young adults were able to increase the stride width of the internal and external leg when turning at higher turning amplitudes, probably increasing their base of support in order to adapt their gait pattern to increased difficulty. This adapting strategy is lost in elderly adults (Madrid et al., 2023).

The gait pattern of young adults during straight-line walking maximizes vertical and antero-posterior stability while maintaining suboptimal mediolateral stability (Latt et al., 2008). At higher turning amplitudes speed decreases, suggesting an optimization of mediolateral stability. This should be addressed in a future study.

In both walking speed conditions, the spatial parameters shorten with greater turning amplitudes while temporal parameters are highest during 180° turn. These insights are important in order to design future studies with a specific focus.

At lower speed, the double support duration (a temporal parameter increasing stability) as well as the single support duration (a parameter reducing stability) increased. Probably because temporal parameters were increased volitionally and not constrained to enhance stability. In contrast in elderly subjects, only the double support duration is increased (Madrid et al., 2023).

Certain aspects of this study warrant discussion. Firstly, the analyses were conducted on the mean and variability of the spatiotemporal parameters within each trial. This method had the benefit of eliminating differences between trials (for instance, initiating with the left or right leg or varying number of steps), thereby mirroring the analysis typically performed in straight-line walking studies. However, this approach did not allow for characterization by turning phases, necessitating further research to examine the biomechanics of turning at specific events or periods during the turning maneuvers. Secondly, turns at slower walking speeds were analyzed. In order to gain more insight into the effect of difficulty on spatiotemporal parameters during turning, it would be interesting to study a more challenging task. For example, it could involve turning while performing a dual task. Thirdly, while the sample size may seem small, it's important to note that the statistical analysis was based on 540 trials, and the statistical power was enhanced by the use of a repeated measure design, incorporating within-subject factors. The sample size was also appropriate given the exploratory objectives of the study. Lastly, future studies involving subjects with pathological conditions will be necessary to further our understanding of turning biomechanics.

## Conclusions

This research broadens our comprehension of turning biomechanics in relation to walking speed. Young adults volitionally reducing their walking speed while turning at different turning amplitudes significantly decreased their cadence and spatial parameters while increasing their temporal parameters. At a slower speed, the variability of some spatial parameters was decreased, whereas the variability of some temporal parameters was increased.

Specifically, cadence was found to be a key difference of the slower turning pattern. Cadence might be a pace gait constant synchronizing the rhythmic integration of several inputs to coordinate an ordered gait pattern output. Volition might up-regulate or down-regulate this pace gait constant (i.e., cadence), which creates the feeling of modulating walking speed.

## Data availability statement

The raw data supporting the conclusions of this article will be made available by the authors, without undue reservation.

## Ethics statement

The studies involving humans were approved by Commission d'éthique Clinique CHUV Lausanne. The studies were conducted in accordance with the local legislation and institutional requirements. The participants provided their written informed consent to participate in this study.

## Author contributions

JM: Conceptualization, Data curation, Formal analysis, Investigation, Methodology, Resources, Software, Validation, Visualization, Writing – original draft, Writing – review & editing. LB: Methodology, Writing – review & editing. MS: Methodology, Writing – review & editing. BU: Conceptualization, Investigation, Methodology, Software, Supervision, Writing – review & editing. BJ: Funding acquisition, Project administration, Resources, Supervision, Writing – review & editing. JF: Conceptualization, Funding acquisition, Project administration, Resources, Supervision, Writing – review & editing. DB: Conceptualization, Funding acquisition, Project administration, Resources, Supervision, Writing – review & editing.
